# Syrian Medical Practice

**Published:** 1846-07

**Authors:** 


*Syrian Medical Practice. Mode of Remunerating Medical Practitioners.*—The physician of the late Lady Hester Stanhope gives the following account of the state of medical practice in Syria. He was called in to attend the son of Ahmed Bey. "There were three physicians present—a Turk, and two Christians. The boy was about thirteen or fourteen, ugly, of diminutive stature, and somewhat humpbacked. He was labouring under anasarca, consequent on a long intermittent fever. After examining him, I said I saw no reason to doubt the possibility of curing him. I was then asked how I would do it, which I declined telling; for I had no one but my servant for an interpreter, and the little Italian which he knew made it impossible to explain my intention clearly. The Bey told me that nothing had been left unattempted which the faculty of the city could think of. His son had been sewed up in a sheep's skin, fresh from the warm carcase: he had taken pills made of powdered pearl: he had lived for six days on nothing but goats' flesh: he had had pigeons' skins put hot to his feet, but all had proved unavailing! I merely observed, that these remedies might have much merit in them, but that the practice of medicine in England was somewhat different, and if he wished me to prescribe, my first condition was that I should not be controlled by any body. After some conversation, I went away. About three hours afterwards I was summoned again, and desired to act as I chose. I was fortunate enough to restore the boy to perfect health; and the father signified to me that he would thank me in the Eastern way. On an appointed day, I was conducted to the vestibule of the bath, where Suliman Bey, attended by half a dozen servants, awaited my coming. We undressed and entered the bath, having each a silk apron on. About an hour was consumed in the ceremonies of shaving the head, washing, depletion, &c., after which he returned to the dressing-room, where we were enveloped in embroidered napkins, and lay down to repose. Pipes, coffee, and sherbets were served. When it was time to dress, the Bey ordered a page to invest me with the dress of honour, which had been prepared for me. It consisted of an entire suit of Turkish clothes, with a pelisse and three pieces of Damascus silks, not made up. The whole might be valued at fifty pounds."—*London Med. Gaz.*

